# The Italian document: decisions for intensive care when there is an imbalance between care needs and resources during the COVID-19 pandemic

**DOI:** 10.1186/s13613-021-00888-4

**Published:** 2021-06-29

**Authors:** Luigi Riccioni, Francesca Ingravallo, Giacomo Grasselli, Davide Mazzon, Emiliano Cingolani, Gabrio Forti, Vladimiro Zagrebelsky, Riccardo Zoja, Flavia Petrini

**Affiliations:** 1 Anesthesia and Intensive Care, San Camillo–Forlanini Hospital, Circonvallazione Gianicolense, 87, 00152 Rome, Italy; 2 Legal Medicine, Department of Medical and Surgical Sciences, University of Bologna, Bologna, Italy; 3 Department of Anesthesia, Intensive Care and Emergency, Fondazione IRCCS Ca’ Granda Ospedale Maggiore Policlinico, Milan, Italy; 4 UOC Anesthesia and Intensive Care, Belluno Hospital, Belluno, Italy; 5 Criminal Law, Università Cattolica del Sacro Cuore, Milan, Italy; 6 Laboratorio dei Diritti Fondamentali, Collegio Carlo Alberto, Turin, Italy; 7 Institute of Legal Medicine, Department of Biomedical Sciences of Health, University of Milan, President of SIMLA, Milan, Italy; 8 President of SIAARTI, Chieti, Italy

**Keywords:** COVID-19, Pandemic, Resource allocation, Triage

## Abstract

**Background:**

In early 2020, the Italian Society of Anesthesia Analgesia Resuscitation and Intensive Care (SIAARTI) published clinical ethics recommendations for the allocation of intensive care during COVID-19 pandemic emergency. Later the Italian National Institute of Health (ISS) invited SIAARTI and the Italian Society of Legal and Insurance Medicine to prepare a draft document for the definition of triage criteria for intensive care during the emergency, to be implemented in case of complete saturation of care resources.

**Methods:**

Following formal methods, including two Delphi rounds, a multidisciplinary group with expertise in intensive care, legal medicine and law developed 12 statements addressing: (1) principles and responsibilities; (2) triage; (3) previously expressed wishes; (4) reassessment and shifting to palliative care; (5) collegiality and transparency of decisions. The draft of the statements, with their explanatory comments, underwent a public consultation opened to Italian scientific or technical-professional societies and other stakeholders (i.e., associations of citizens, patients and caregivers; religious communities; industry; public institutions; universities and research institutes). Individual healthcare providers, lay people, or other associations could address their comments by e-mail.

**Results:**

Eight stakeholders (including scientific societies, ethics organizations, and a religious community), and 8 individuals (including medical experts, ethicists and an association) participated to the public consultation. The stakeholders’ agreement with statements was on average very high (ranging from 4.1 to 4.9, on a scale from 1—full disagreement to 5—full agreement). The 4 statements concerning triage stated that in case of saturation of care resources, the intensive care triage had to be oriented to ensuring life-sustaining treatments to as many patients as possible who could benefit from them. The decision should follow full assessment of each patient, taking into account comorbidities, previous functional status and frailty, current clinical condition, likely impact of intensive treatment, and the patient's wishes. Age should be considered as part of the global assessment of the patient.

**Conclusions:**

Lacking national guidelines, the document is the reference standard for healthcare professionals in case of imbalance between care needs and available resources during a COVID-19 pandemic in Italy, and a point of reference for the medico-legal assessment in cases of dispute.

## Background

The SARS-CoV-2 pandemic has put the Italian National Health Service (Servizio Sanitario Nazionale, SSN) under extraordinary pressure, leading in some regions and in some periods to imbalances between demand and supply of care [1, 2].

At the beginning of the pandemic, there were 5.179 intensive care unit (ICU) beds (8.8 beds/100,000 inhabitants) in Italy, with wide variations among the 21 Italian regions. During the first wave, in some regions, the threshold of 30% of ICU beds occupied by COVID-19 patients as the maximum of ICU admissions per week was exceeded (in December 2020, after implementation of resources by the government, the number of ICU beds increased to 8.765).

In expectation of worsening of the imbalance between the need for ICU admission and the actual availability of resources (beds, ventilators, specialized staff) caused by the increasing number of people with COVID-19 with acute respiratory failure, on 6 March 2020 the Italian Society of Anesthesia, Analgesia, Resuscitation and Intensive Care (SIAARTI) issued *Clinical Ethics recommendations for the allocation of intensive care treatments, in exceptional, resource-limited circumstances* [3]: in order to maximize the benefits for the greatest number of people, it was recommended to give priority to patients most likely to survive and secondarily to those who may have the most years of life saved. The guiding priority criteria were age, comorbidities, and functional status.

To our knowledge, these recommendations were the first to be published anywhere on the topic of ICU resource allocation during the COVID-19 emergency. They have been referenced in numerous publications and mentioned in other similar recommendations [4–8] and have provoked extensive debate in Italy, which is still ongoing [9]. Indeed, even in the extraordinary conditions of the pandemic, the four principles of medical ethics (autonomy, beneficence, non-maleficence, justice) remain the landmarks for every clinical decision.

On 9 July 2020, the Italian National Institute of Health (ISS) invited the Presidents of SIAARTI and the Italian Society of Legal and Insurance Medicine (SIMLA) to prepare a draft document defining the triage criteria for intensive care in the pandemic emergency to be implemented in case of complete saturation of available resources. The draft document was intended as a basis for analysis and sharing with other scientific and technical-professional societies, associations and other relevant stakeholders.

After a public consultation, the final version of the document was published on the ISS National Center for Clinical Excellence, quality and safety of care (CNEC) website on 13 January 2021 in the section dedicated to “best clinical practices”. In accordance with Italian Law 24/2017, professional activities in Italy are legally assessed on the basis of their compliance with guidelines published on the ISS CNEC website and, in the absence of these, with best clinical practices like those suggested in this document.

This article summarizes the document’s scope, development and content.

## Objectives

The general objective of the document was to provide healthcare professionals with a tool for responding appropriately to the emergency due to the current COVID-19 pandemic—which potentially affects the health of the entire community—in the event of an imbalance between healthcare demand and available resources, with particular reference to intensive care resources.

Specific objectives were the following:

- to provide healthcare professionals with shared criteria for the admission, stay and discharge of patients in the high-intensity care levels of hospitalization, as well as to guide towards the most appropriate treatment of patients in medium and low-intensity care;
- to ensure the transparency of healthcare professionals' choices through a clear explanation of decision-making criteria;
- to preserve trust between people, healthcare workers and the national health system during the emergency.

## Ethical and legal principles underlying the document

According to the ISS request, both ethical and legal principles should be at the basis of the document.

### Principles established by the Italian constitution and legislation

- Right to health—art. 32 of the Italian Constitution: “The Republic safeguards health as a fundamental right of the individual and as a collective interest and guarantees free medical care to the indigent”.
- Principle of equality and equal social dignity—art. 3 of the Italian Constitution: “All citizens have equal social dignity and are equal before the law, without distinction of sex, race, language, religion, political opinion, personal and social conditions”.
- Duty of solidarity—art. 2 of the Italian Constitution: “The Republic recognizes and guarantees the inviolable rights of the person, both as an individual and in the social groups where human personality is expressed. The Republic expects the fundamental duties of political, economic and social solidarity to be fulfilled”.
- Universality and fairness—art. 1 of Italian Law No. 833/1978: “The national health service is made up of a complex of functions, structures, services and activities aimed at the promotion, maintenance and recovery of physical and mental health of the entire population without distinction of individual or social condition and in a manner that ensures the equality of citizens with respect to the service”.
- Respect for self-determination—art. 32 of the Italian Constitution: “No-one can be obliged to undergo any health treatment except under conditions set by law. The law may not in any circumstances violate the limits imposed by respect for the human person”; Italian Law No. 219/2017 on “Regulations on informed consent and advance treatment dispositions”.

### Ethical duties (according to the Hasting Center [10])

- Duty to care—a duty that requires physicians to observe the function of guarantee.
- Duty to guide—how to allocate resources is an ethical duty of decision-makers within the health care system.
- Duty to plan—health care leaders' duty to plan for the management of foreseeable ethical challenges during a public health emergency.
- Duty to safeguard—duty to protect health care workers, the population, and especially vulnerable individuals.

## Methods

A Working Group (WG) was formally appointed on 20 July 2020. The members were experts in anesthesia and intensive care (LR, GG, DM, EC, FP), legal medicine (FI and RZ, who had expertise in clinical ethics and medical malpractice) and law (GF and VZ) designated on the basis of their expertise in the field of interest identified through the PESTEL (Politics, Economy, Society, Technology, Environment, Law) [11] method of analysis.

In a web-conference, using the SWOT analysis (Strengths, Weaknesses, Opportunities, Threats) [12], the WG identified five areas of interest from which to draw questions and define statements to manage the imbalance between demand for care and resources.

These are the five areas: (1) Ethics, (2) Law, (3) Organization, (4) Management of incoming patients, and (5) Management of patients in hospital. Within the areas of interest, several questions were identified as preliminary to definition of the statements that serve as the WG answers. The Delphi methodology [13] was adopted to systematically build the consensus among the WG experts. An initial version of the statements was drafted following the WG meetings, which took place online, as well as numerous discussions via e-mail. Subsequently, following the Delphi methodology, the WG members were asked to express their agreement according to a 5-point Likert scale (from 1 = full disagreement to 5 = full agreement) on the title of the document and on 13 statements, and to provide any comments.

Based on the results of this first round, the statements with most differences in agreement were edited and two statements were merged. The WG members were then asked again to express their level of agreement on the title and the 12 statements (using the same rating scale). The second round gave a high level of agreement (full agreement/agreement) for 8 of the 12 statements. One WG member indicated disagreement regarding the wording of statements 2 and 12, while, respectively, one WG member and two others indicated "indifferent" as the levels of agreement for one of the parameters in statement 6 and for statement 7, respectively. All statements were then accepted and in the following month the WG drafted explanatory comments for them all, in five sections: (1) principles and responsibilities; (2) triage; (3) previously expressed wishes; (4) reassessment and shifting to palliative care; and (5) collegiality and transparency of decisions.

The draft document underwent a public consultation from 19 November to 10 December 2020. This consultation was organized by the ISS CNEC in accordance with its guidelines. The ISS CNEC promoted the consultation through its website (https://snlg.iss.it/?p=3272) and the consultation announcement had broad echo in traditional and online newspapers and on associations’ and scientific societies’ websites. All the about 300 scientific or technical-professional societies recognized by the Italian Ministry of Health (https://www.salute.gov.it/imgs/C_17_pagineAree_4834_5_file.pdf) and those recognized by the ISS CNEC as possible other stakeholders (i.e., “associations and representatives of citizens, patients and family members/caregivers; institutions and religious communities; industry; national and regional public institutions; universities; public and private research institutes”) could participate in the public consultation by registering on the ISS website. All stakeholders were invited to rate their degree of agreement with each statement using a Likert scale from 1 (full disagreement) to 5 (full agreement), and to make any comments. Finally, those interested in the topic who could not register on the website (e.g., healthcare providers, lay people, other associations) could address their comments to the WG by writing to ricerca@siaarti.it.

## Results

The statements are summarized in Table 1.

**Table 1:** Summary of the statements

***Principles and responsibilities***
1. The increase in demand for health care (at the three levels of hospitalization: ordinary, semi-intensive, intensive), due to a situation such as the pandemic, does not reduce the necessary adherence, as regards the protection of health, to the constitutional and founding principles of the National Health Service and to deontological principles, particularly universality, equality (non-discrimination), solidarity and self-determination
2. It is the responsibility of the healthcare organization, at every decisional level, to adapt the response in terms of logistics, technological and human resources to face the crisis
3. In the event of an imbalance between the need for and the supply of care, the healthcare organization is always responsible locally for all the organizational strategies aimed at providing patients with the appropriate treatment (increase in the number of beds in both ordinary and intensive care, increase/redistribution of human and technological resources, implementation of the system for transferring patients between healthcare facilities). Healthcare professionals shall—as effectively as possible—report any shortcomings that make it impossible to implement these strategies to the competent institutions
***Triage***
4. At every level of intensity of care, should care resources become saturated, making it impossible to guarantee each sick person the recommended treatment, it is necessary to resort to triage rather than to a “first come, first served” or random (lottery) criterion
5. The purpose of intensive care triage is, in accordance with the above principles, to ensure life-sustaining treatments to as many patients as possible who may benefit from them
6. Triage should be based on defined clinical–prognostic parameters that are as objective and shared as possible. The assessment, aimed at stratifying the likelihood of overcoming the current critical condition with the support of intensive care, should be based on an overall evaluation of each individual patient, using the following parameters:
Number and type of comorbidities
Previous functional status and frailty relevant to the response to care
Severity of the current clinical condition
Presumable impact of intensive treatments, also taking the patient's age into account
The patient's wishes with regard to intensive care, which should be explored as early as possible in the initial triage stage.
7. Age should be considered as part of the global assessment of the patient and not on the basis of pre-set cut-offs
***Previously expressed wishes***
8. If a patient lacks decision-making capacity, a careful check should be made for any previous wishes expressed through advance directive or shared care planning. Shared care planning should be offered to all patients likely to require intensive care in the future
***Reassessment and shifting to palliative care***
9. All access to care should be scrutinized on the basis of daily reassessment of the clinical indication, goals of care and proportionality
10. Patients for whom intensive care is not possible must receive—in relation to their clinical and healthcare related situation and their wishes—the most appropriate treatments, including palliative care, which must always be guaranteed at all levels of hospitalization and in all care settings
11. Should a patient not respond to treatment or severely worsen the decision to discontinue intensive care (withdrawing futile treatments) and to shift to palliative care must not be postponed
***Collegiality and transparency of decisions***
12. Compatibly with human resources and actual circumstances, decisions should not be left to a single provider, but should be the result of collective evaluation by the medical and care team, which may, if necessary, also call on outside professionals (for a “second opinion”). The decision to limit intensive care must be adequately justified reported and documented in the medical record

Eight stakeholders (including two medical and two scientific nursing societies, a local heath trust, an association of bioethicists, a religious community, and the ethics committee of a scientific hospital center) participated in the public consultation. The list of stakeholders and their average ratings for each statement was reported on the ISS CNEC website (https://snlg.iss.it/wp-content/uploads/2021/01/Risultati-conslutazione-pubblica-doc-Triage_SIAARTI-SIMLA.pdf). In addition, eight e-mails with comments and criticisms were directly addressed to the WG by experts in palliative care (two), anesthesiology and intensive care (two), legal medicine, oncology, ethics and by an association of retired doctors and widows.

Almost all stakeholders, whose agreement with each statement was on average very high (ranging from 4.1 for statement 7 to 4.9 for statement 9), and those who participated on their own initiative expressed substantial appreciation for the document. The main criticisms focused on the following topics: the inclusion of the age-adjusted Charlson index among the possible triage tools, especially for the importance the index gives to age and oncologic comorbidity; the need to emphasize the role of palliative care experts; and the lack of thorough analysis of ethical issues. One scientific association of critical care nurses strongly disapproved the triage statements (rating statements from 5 to 7 with “full disagreement”) and disagreed with statement 12. These were the only cases of disagreement expressed by the stakeholders. Among those who addressed their remarks directly to the WG, the association of retired doctors and widows expressed legal concerns, sending a letter drafted by a lawyer who questioned the legality of the triage per se.

The WG thoughtfully considered all these comments and in some cases modified the statements wording, but not their content. Finally, the WG submitted the final revised version of the document to the ISS CNEC, which published it without asking the WG for any further revisions.

## Discussion

### Principles and responsibilities (Statements 1 to 3)

Even when there is an extraordinary imbalance between the need and supply of care, the primary and fundamental right to health must be ensured, as an essential element of human dignity, in compliance with the principles of ethics and justice and, therefore, in accordance with the universal egalitarian criterion of access to care. Hence the necessary adherence to the following main principles:

- Equality: everyone has the full and unconditional right—regardless of age, social condition, ethnicity, etc.—to receive the best available healthcare; professionals have the deontological obligation to oppose any form of discrimination in access to care;
- Social solidarity: this guarantees attention to those who are most vulnerable and implies solidarity among everyone, taking account of the fact that the health of an individual cannot be considered independently from the health of the community and vice versa (article 32 of the Italian Constitution);
- Self-determination: respect for human dignity and freedom is ensured in the protection of health.

The observance of these principles implies first of all the responsibility, at the institutional level, to adopt strategies aimed at preventing as far as possible, with adequate allocation of health resources (and an active search for or increase of these resources, if necessary), serious shortfalls in resources. Secondly, healthcare professionals on the frontlines must implement organizational strategies, in addition to their clinical commitment hence including extraordinary organizational strategies to ensure that each patient receives appropriate treatment. To this end, close cooperation between clinicians and other relevant professionals and institutional representatives is essential. In fact, reporting critical situations may start directly from those engaged in the field, but possible solutions should be offered by an organization of some sort that provides for involvement at every level (local, regional, national) of anyone who has the possibility and the duty to make an effective contribution.

Once it has been verified that it is impossible to adapt the healthcare response to the scenario produced by the crisis, for the sake of transparency towards both healthcare workers and the community this must be clearly and explicitly stated at all institutional levels.

### The triage (Statements 4 to 7)

When there is an imbalance between the need for care and healthcare resources, treating sick people according to the “first-come, first-served” approach would not be fair. This rule, unlike the egalitarian approach, risks being discriminatory because—as suggested by one stakeholder during public consultation—the time when the person goes to hospital is likely to be influenced by economic conditions, social context and individual possibilities of the patient or his family. It is therefore essential to apply a system of triage, meaning an assessment aimed at establishing which patients should be given priority for treatment.

In intensive care, it is a matter of making an overall comparative assessment of the patients’ conditions, not with the aim of establishing who is more serious or in greater need of care, but who is most likely (or least likely) to overcome the current critical condition with intensive care, with reasonable life expectancy once out of the ICU that is, short-term survival (months) after discharge from the hospital.

The proposed triage criteria here have no predefined hierarchy and should not be seen as unquestionable, but should be balanced and viewed in the light of each clinical condition, where one criterion or more may become more important and thus predominate in the clinical decision. In the overall assessment of a patient, assessment tools that can at least serve as an aid for comparative prognostic framing may include the Charlson index [14–16], the Clinical Frailty Scale [17–22,], and, only in patients with COVID-19, the Coronavirus Clinical Characterisation Consortium mortality score [23–26]. When appropriate, these tools, useful simply for general orientation, can enable healthcare providers to talk within the team and with patients and their family or legal representatives on the basis of recognized prognostic indicators, aware that there is currently no single tool, or set of tools, that can replace a global clinical assessment. It is therefore inappropriate to make the outcome of intensive care triage dependent on the score given by any tool or algorithm, even those proposed or used in other countries.

In subjects with previous comorbidities, the severity and stage of illness should be assessed on the basis of objective criteria and parameters [27].

The patient's opinion and preferences regarding treatment are essential for deciding the proportionality of care. The patient's wishes regarding treatment should therefore be explored at an early stage, to avoid triaging people who do not want intensive care.

Age in itself is not a criterion for deciding which patients are most likely to benefit from intensive care and therefore cannot be used by establishing age cut-offs in the triage. Other conditions being equal, age may play a role in assessing a patient because the outcome of intensive treatments is likely to worsen with age [28–30].

The triage process should be documented in the medical record, possibly using a specific ad hoc form [31]. The patient, any legal representatives, and family members should be informed of the triage outcome and the estimated likelihood of recovery if admitted to the ICU.

### Previously expressed wishes (Statement 8)

If the patient is not able to give consent, any wishes expressed previously must be explored during triage, consulting the database of advance directives too. If the patient has expressed a wish contrary to the treatments indicated that person’s will must be respected, unless there are circumstances established by law (Law 219/2017, art. 4). A check must also be made for any shared care plan consulting the patient’s health record and contacting the attending physician (general practitioner and/or specialist).

If a legal representative or trustee is present, the physician will contact them.

All patients likely to need life-sustaining treatment should be offered shared care planning. The patients and, with their consent, their family members should be adequately informed “in particular about the possible evolution of the current pathology, what the patient can realistically expect in terms of quality of life, the clinical possibilities for intervention and palliative care” (Law 219/2017, art. 5).

### Reassessment and shifting to palliative care (Statements 9 to 11)

Re-evaluation of the current clinical indication, goals and proportionality of care must be entered in the medical record on a daily basis. Appropriateness and proportionality of care are the ethical and professional prerequisites for any treatment, and the continuation of inappropriate or disproportionate treatment is not permitted.

Should it be decided to limit intensive care, due to lack of clinical appropriateness, proportionality or as a result of comparative assessment during triage, the physician must inform the patient, when possible, and family members and legal representatives about the appropriate treatments for each single case, to be provided in a non-intensive setting, at home when possible.

If during daily reassessment it is found that the indications for treatment no longer exist or that the treatment is futile in relation to the therapeutic objectives, the patient (if capable of receiving the information) and his/her family or legal representatives must be informed immediately. The need to shift treatment towards palliative care, including palliative sedation if necessary, should be discussed with them. Palliative care should be readily available in the ICU or some other appropriate setting. At admission to the ICU, the patient (when capable of receiving the information), legal representatives and family members should be informed of the possibility of shifting treatment if it becomes futile.

### Collegiality and transparency of decisions (Statement 12)

The decision to limit intensive care, whether at triage or later, should never be left to a single physician, given the ethical and psychological enormity of any such decision. In usual clinical practice, the decision to discontinue futile treatments is generally shared by the whole medical and nursing team. This model of decision-making and sharing should be adopted for all decisions to limit intensive care, making use also of the possibilities offered by telemedicine under the conditions of physical isolation imposed by the pandemic. The decision should be even more thorough and shared, if possible, in the case of withdrawal of ongoing treatments. If this is not possible, the physician should describe, in the medical record, the circumstances that prevented a team decision, including the fact that there was not enough time for a collective decision.

A “second opinion” may be required in situations of particular uncertainty or disagreement within the team.

All decisions must be duly justified, documented in the medical record and communicated promptly and understandably to the patient, when capable of receiving the information, to legal representatives and to family members. Even in these times of overloaded healthcare services, communication must remain a priority, and adequate time and space must be allowed.

## Conclusions

Triage and the triage criteria proposed in this document were applicable from the period during the COVID-19 pandemic when a shortage of care resources arose, and they remain applicable as long as extraordinary resource allocations are necessary. Intensive care triage is applicable to all patients who need intensive care, regardless of the underlying clinical condition; it is therefore an independent process following that of assessing the appropriateness and proportionality of intensive care. Triage does not apply to patients already receiving intensive care.

Lacking national guidelines, the document is the reference standard for healthcare professionals’ in case of imbalance between care needs and available resources during a COVID-19 pandemic in Italy, and a point of reference for the medico-legal assessment in cases of dispute.

## Limitations

The restricted number of experts and expertise involved in the WG might be considered a limitation, but they were selected as representatives of the scientific societies appointed by the ISS. Furthermore, the SIAARTI was involved in the national Technical Scientific Committee on the COVID-19. Finally, the judgements and suggestions of the stakeholders boosted the involvement of experts.

The most important limitation is the lack of strong evidence from the scientific literature about the clinical criteria and prognostic factors for the comparative evaluation of patients.

## Data Availability

Not available.
